# Chronic Effects of Strength Training Vs. Hydro Aerobics on Functional and Cardiorespiratory Ability in Postmenopausal Women

**DOI:** 10.2478/hukin-2014-0090

**Published:** 2014-11-12

**Authors:** Giovanni S. Novaes, Jefferson S. Novaes, José Vilaça-Alves, Gabriel Costa e Silva, Nuno D. Garrido, Hélio Furtado, Victor M. Reis

**Affiliations:** 1 Research Center for Sports Sciences, Health & Human Development (CIDESD), Vila Real, Portugal.; 2 School of Sports and Physical Education, Rio de Janeiro Federal University - UFRJ - Rio de Janeiro, RJ, Brazil.; 3 Department of Sports Sciences, Exercise and Health, University of Trás-os-Montes and Alto Douro (UTAD), Vila Real, Portugal.; 4 Laboratory of Physiology and Human Performance, Department of Physical Education and Sports, Federal Rural University of Rio de Janeiro - UFRRJ - Rio de Janeiro, RJ, Brazil.; 5 Castelo Branco University, Rio de Janeiro,RJ, Brasil.; 6 Secretaria Municipal de Envelhecimento Saudável e Qualidade de Vida, Prefeitura do Rio de Janeiro, Brasil. .

**Keywords:** strength training, hydro gymnastics, functional aptitude, cardiorespiratory fitness, women, menopause

## Abstract

The current study aimed to compare the effects of two exercise programs (Strength Training and Hydro Gymnastics) on the functional and cardiorespiratory abilities of Portuguese postmenopausal women. The study population consisted of 38 volunteers (age: 66.9 ± 6.1 years, body mass: 73.70 ± 10.38 kg, and body height: 1.55 ± 0.10 m). Subjects were randomly divided into two experimental groups and one control group: one group performed 24 weeks of strength training (GST; n = 14), another performed 24 weeks of hydro gymnastics (GH; n = 17) and a control group (CG; n = 7), where the subjects continued with their regular daily activities without involvement in any physical exercise program. Three assessments were performed: before the beginning of the program, 12 weeks after the start of the training program and 24 weeks after the start of the program. To assess the functional ability of the participants, several tests proposed by Jones and Rikli (2002) were performed. To evaluate the cardiorespiratory ability of the participants, a modified treadmill Bruce test was applied. Significant differences (p<0.05) were found between the two training methods in the tests, which primarily demanded muscular strength. Body mass and the body mass index showed significant differences during the three stages of assessment in the GST group (p<0.05). With respect to the values that represent the variables of cardiorespiratory ability, positive and significant changes were observed in the two experimental groups. It was concluded that both exercise programs promoted improvements in some indicators of the functional and cardiorespiratory abilities of Portuguese postmenopausal women.

## Introduction

The aging process varies widely among people and is influenced by both lifestyle and genetic factors (Nieman, 1999). In this case, the functional autonomy, also called the functional capacity, presents one of the most important concepts in relation to health, physical fitness and quality of life. Functional capacity is also a determining factor in the analysis of the effects of aging because it may be associated with the decline in ability to perform activities of daily living more so than the actual chronological age (Matsudo, 2001).

The aging process tends to negatively influence physiological and motor functions (Rockwood et al., 2005). Several studies focused on understanding the mechanisms involved in the aging process have demonstrated the importance of an active lifestyle at all stages of life. The adoption of physical exercise in the daily lives of elderly people has mainly focused on health promotion (Araujo and Ceolim, 2007; Häkkinen et al., 2010; Harris et al., 2009; Henderson and Ainsworth, 2003).

The recommendation of exercise programs for seniors should seek to improve their neuromuscular fitness, with a focus on the prevention of falls and improvement of balance, posture, and cardiorespiratory fitness. These exercises, when combined with neuromuscular and stretching exercises, can provide elderly individuals with strength and joint mobility, which favors significant effects on health and fitness (Seguin et al., 2008; Simons and Andel, 2006). Blankevoort et al. (2010), in a review study, concluded that the implementation of exercise programs for the elderly provides improvements in the perception of quality of life and increases cardiorespiratory and neuromuscular fitness. Additionally, the improvement of mental well-being, which promotes significant changes in self-esteem and autonomy, is reported as effect of the implementation of exercise programs in the elderly population (Beswick et al., 2008).

Strength training programs are becoming common in recommendations of physical training for the elderly in order to improve their neuromuscular capacity, functional autonomy and self-esteem (Kimura et al., 2010). Additionally, hydro aerobic programs present benefits in terms of functional autonomy (Beswick et al., 2008), neuromuscular capacity (Colado et al., 2009a; Bento et al., 2012; Bergamim et al., 2013; Colado et al., 2013) and cardiorespiratory fitness (Santana and Maia Chaves, 2009; Colado et al., 2009b).

Similarly, water-based activities improve the quality of life by reducing psychopathological symptoms (Schuch et al., 2013), which are common in postmenopausal women. Thus, exercise is of fundamental importance for the elderly in order to maintain active lifestyle and a good quality of life. However, while studying the literature on the subject, some gaps in knowledge were observed about the chronic effects of strength training and hydro aerobics on cardiorespiratory and functional fitness.

Therefore, to investigate the benefits developed by different exercise programs aiming at promotion of optimal health for the elderly, the present study attempted to compare the effects of two exercise programs (strength training vs. hydro aerobics) on the functional and cardiorespiratory fitness in postmenopausal women over a period of 6 months.

## Material and Methods

### Sample

The sample consisted of 38 postmenopausal elderly, Caucasian women (age: 66.9 ± 6.1 years, body mass: 73.70 ± 10.38 kg; body height: 1.55 ± 0.10 m) divided into three following groups: 1) a group where a strength training program was applied (GST, n = 14), 2) a group where a hydro aerobic program was applied (GH, n = 17) and 3) a control group (CG, n = 7), where subjects continued with their daily tasks without participation in any type of planned physical exercise. The sample inclusion criteria were as follows: female, older than 55 years of age, and post-menopausal. All subjects completed the Par-Q test and anamnesis that included questions about age, physical activity habits, heart problems, chest pain, lack of balance and the use of medication for blood pressure or heart problems.

### Procedures

On the first visit to Foz do Cávado - Esposende Pools, the procedures and the fundamentals of the study were explained to all subjects, and any questions the participants had were answered. After all questions were clarified, the subjects gave their written consent according to the Declaration of Helsinki, 1975, for their participation in this study. Subsequently, we performed the measurements for body height (BH) using a scale stadiometer with an accuracy of 0.1 m (Country Technology ™, model 67031, Gays Mills, WI, USA), body mass (BM) using a brand balance (Tanita Corporation Model: BF-562, Illinois, USA) and an assessment of elderly functional autonomy using tests proposed by Rikli and Jones (2002). The functional autonomy of the upper and lower limbs was evaluated using the following tests: sit and stand on a chair for 30 s (SS), forearm flexion for 30 s (FF), sit and reach (SR), get up and walk backwards 2.44 m and sit (GUW) and reaching behind the back with the hands (RBB). We assessed the training load for the subjects who performed the strength training (ST) test by converting 7–10 maximum repetitions (MR) to 1RM through the formula proposed by Knutzen et al. (1999). The exercises included in the evaluation were as follows: squat with dumbbells (SD), rowing back with dumbbell (RD), seated leg flexion (SLF), vertical chest press (VCP), unilateral dumbbell (UD) with both arms, shoulder press with dumbbells (SPD), dumbbell biceps curl (DBC), and triceps extension on the handle top (TE).

Cardiorespiratory fitness was assessed by a submaximal test using the adjusted Bruce protocol (Table 1), carpet crane model 5000 (AC SCIFIT Corporate, UK) and a k4 gas analyzer (Cosmed, Rome, Italy). Before each test, the calibration of the k4 analyzer was made according to the manufacturer’s instructions: a) calibration of turbine syringe with 3 liters, 2) calibration with a gas mixture (16% O2 and 5% CO2), 3) calibration of the delay, and 4) calibration with ambient air. During the protocol, expired gases were measured breath-by-breath and recalculated over an average of 20 seconds. The test was stopped when the subjects presented a heart rate (HR) of more than 80% of the HR maximum, which was calculated using the following formula: HRmax = 220 - age. In this protocol, variables of submaximal oxygen consumption (VO_2sub_) and the total duration of the test (T_totaltest_) were excluded. The systolic and diastolic blood pressure was measured with an aneroid sphygmomanometer OMRON model M6 (OMRON Healthcare Co. Ltd) when the subjects had been seated for 10 minutes. Additional measurements were taken in a quiet and controlled environment with respect to the noise level, visual stimulation and temperature, which was measured with a thermometer-type hygrometer (portable model, “Testo 625,” Testo, Germany). All dependent variables in this study were observed during three separate assessment times: pre-test (T1), 12 weeks after the pre-test (T2) and 24 weeks after the pre-test (T3).

The GST group performed strength training (ST) 3 days per week for 24 weeks. Before the start of each session, 5 minutes were allowed for the subjects to perform coordinated movements with music and for joint mobility. In the first two weeks, the subjects performed 8 exercises (SD, RD, SLF, VCP, abdominal crunch (AC), SPD, extension of the back with a rod held by the hands (EBH) and DBC); in the subsequent 10 weeks, the UD and TE exercises were added.

In all sessions, the following routine was followed: three sets of each exercise, with 10 repetitions in the first session, 8 in the second and 12 in the third, except for the exercises AC and VCP, for which subjects always performed 20 repetitions per set of exercises; a load of 75, 80 and 60% of 1RM in the 1^st^, 2^nd^ and 3^rd^ sessions per week, respectively, with the exception of the exercises AC and EBH, which were always performed with body weight; 1 min rest periods between sets of exercises were used; 1 min, 30 s and 2 min for each set in the 1^st^, 2^nd^ and 3^rd^ sessions per week, respectively, except in the DBC and EBH exercises, for which all sessions lasted 1 min.

In the first two weeks, the goal was to create muscle, joint and tendon adaptation to the ST. The remaining 10 weeks were dedicated to the development of functional autonomy of the elderly, mainly through structural adaptations in the muscle. During these 12 weeks, the training load was increased by 5% of the initial 1RM of 3 in 3 weeks, and training was always supervised by qualified personnel with ST experience. In the following 12 weeks, the program aimed at increasing the functional autonomy of the elderly through the conjugation of ST and exercises that tried to mimic daily situations (DS), namely, squat exercise in conjunction with the stand or sit in a chair, chest press exercise (with the vertical support of a wall, flexing the two upper limbs and returning to the upright position only with strength of the upper limbs), adduction of legs (with the simulation of getting into a car using a step; the height of the seat was approximately the height of an automobile seat with two boards on either side of the step equal to the seat height), front elevation of the upper limbs and then extending from the ankle, placing a weight of 1 kg on a shelf (height of a kitchen cabinet), shrugs with dumbbells induced by the flexion of the R portion of the trapezius muscle group followed by bending the elbows with the simultaneous transport of a few grocery bags (each 3 kg), and abdominal and hip flexion followed by VCP.

In the ST that was combined with DS during these 12 weeks, the subjects performed 8 repetitions with a load of 80% of 1RM, with the exception of abdominal exercises with hip flexion and GTE, which were performed with the subjects’ own body weight. The load in these exercises was increased by 5% of 1RM of 3 in 3 weeks. The DS were performed without additional change beyond that which was previously established for 30 seconds. The SF and DS were performed sequentially in each series. The subjects performed 3 sets of each block of exercises, with a 2–3 minute rest between sets.

The GH group performed hydro aerobic training that was divided into segments during the first two weeks: 10 minutes of general activation in which the subjects performed displacement exercises, stationary floating and movement in different directions, and the intensity varied between 120 and 140 beats per minute (bpm); during the most important part of the class, which lasted 30 minutes, the emphasis was on the cardiopulmonary component, which used exercises focused on the lower limbs (LL) and upper limbs (UL), ranging in amplitude for the abdominal muscle group. At this stage, the depth of the water in the pool ranged from no impact (floating) to 1.40 m. The cool-down lasted 5 minutes; the subjects performed stretching exercises individually and in pairs. After the initial two weeks, the general activation increased between the 3rd and 4th week to 15 minutes and consisted of the same exercise, increasing only the impact. Subjects could return to a rested state to stretch on the edge of the pool, where the depth ranged from 1.10 to 1.40 m.

The heart rate was maintained between 120 and 140 bpm. In the 5th and 6th week, general activation lasted 20 and 15 minutes, respectively, and remained high-impact, except for the second day of training of the 6th week; at this time, there was interval training, and the heart rate was maintained between 100–140 bpm. The exercises of the main portion of the class were the same, with the inclusion of interval training; the depth of the water was 1.10–1.40 m. From the 7th to the 24th week, resistance gloves were added; the exercises remained the same with the inclusion of hydro power for both upper and lower limbs.

The impact was always high. Effort was monitored through the use of a heart rate monitor (Polar System) and through the adapted Borg scale that was placed in a location visible to all participants. During the main part of the session, the subjects were asked to adjust their exertion to a level of 4–5 (Borg scale) to achieve approximately 70–85% of the theoretical maximum heart rate. Subjects were asked to follow the rhythm of the music; the perception of effort was coincident with the request of the professional who guided the classes. The classes were always instructed and observed by the same experienced personnel.

### Statistical Analyses

We used a third model (GST, GH and CG) X 3 (pre-test and post-test 1 and post-test 2) of analysis of variance for repeated measures (ANOVA) using an analysis of groups and different times. The post hoc Tukey’s test was also used to identify the differences between groups and moments. We tested the normality, homogeneity and sphericity of all variances and co-variances. No violation of assumptions with the use of this type of statistical method was observed. The level of significance was set at p <0.05.

## Results

With respect to the variables of body mass (BM) and body mass index (BMI), significant differences were observed (μ = 0.184) in BM between the GST and GH groups and in the BMI (F = 3.694, p = 0.036, μ = 0.188) between the GST and GH groups (Table 2).

When we analyzed the values for functional autonomy and cardiorespiratory fitness between T1 and T2, we observed significant positive changes in the GH group with regard to SS and GUW tests and in the GST group with regard to SS, GUW, and FF T_totaltest_. Between T2 and T3, positive and significant changes were observed in the SS tests (F = 10.261, p = 0.00, μ = 0.398) between the GH and GST groups, between the GST and CG groups, and between the CG and GH groups. SR (F = 7.015, p = 0.00; μ = 0.312) between the GH and GST groups and between the GST and CG groups was also significantly different. GUW, FF, Diastolic Blood Pressure (DBP) and T_totaltest_ in the GST group and AM tests, SR, GUW, and DBP T_totaltest_ in the GH group were not significantly different.

There were only positive and significant changes from T1 to T2 and from T2 to T3 in the GUW test in the GH group and the tests SS, GUW, FF, DBC (F = 13.607; p = 0.038, μ = 0.467) between the GH group and the GST group; also significant were the differences between the GST and CG groups, and between the CG, GH and GST groups with respect to T_totaltest_.

When variables related to functional autonomy and cardiorespiratory fitness were compared between groups in the 3 evaluation times, it was observed that in T1, the differences were statistically significant between the GST and CG groups with respect to the SR and GUW tests (F = 8.325, p = 0.00, μ = 0.357) and between the CG/GST groups and the CG/GH groups with respect to FF; the GST group presented more positive test performances than the CG group. At an even earlier time, a comparison of the GH and CG groups showed significant differences between the SR and FF tests, and the GH gave higher performance values compared with the values given by the CG group. In T1, between the GST and GH groups, for each of the variables, we observed statistically significant differences. In T2, the GST group presented significant and higher performance values in the SS, GUW, and FF tests and in the SR and FF tests in relation to the CG and GH groups, respectively. However, in the GH group, we observed significantly higher values than in the CG group for the SS and GUW tests. Finally, at T3, the GST group presented higher and more significant performance values in relation to the GH and CG groups with respect to the SS and FF tests. Significant differences were also observed in the tests for SS, GUW, and FF T_totaltest_ (F = 2.638, p = 0.05, μ = 0.150) between the CG and GST groups. When the same moment of measurement was compared between the GH and CG groups, we observed higher performance values for the SS and GUW tests.

## Discussion

Based on the results of this study, we observed a positive effect at the end of 24 weeks in the groups that performed physical activity (GH and GST), with respect to most indicators of functional autonomy and duration for the adapted Bruce Test. Regarding the indicators of cardiac and vascular capacity in these two groups, only DBP in the GH group and in the GST group and VO_2sub_ in the GST group showed significant changes (p <0.05).

In contrast, we did not observe any significant changes in the CG group in any of the variables in this study. Additionally, after 24 weeks, we found a significant and positive difference in the tests of functional autonomy related to physical strength, particularly in the SS and GUW tests and in the duration of the adapted Bruce test in the GH group relative to the CG group, and also the FF in the GST group.

However, because the tests were related to physical strength capacity and because FF in the GH group did not show significant improvements, we can presume that this result may be related to a smaller likelihood that hydro aerobic activity can promote significant increases in physical strength in the exercises where use of the biceps brachii muscle group can be noted, which was also observed in the study of Tsourlou et al. (2006).

In this sense, Colado et al. (2012) verified the effects of 24 weeks of strength training performed in both water and land environments. The authors noted that training conducted in the water environment had similar positive effects on body composition and physical function in postmenopausal women compared to training in the land environment. However, in tests where the physical strength ability was dominant (FF and SS), significant differences between the subjects in the GH and GST groups were observed, with the GH group presenting performance values higher than those of the GST group. Similarly in our study, at the end of the 12-week intervention, the ST was sufficient to improve the performance in the SS and GUW tests and in the duration of the adapted Bruce test; in hydro aerobic training, the only improvements were observed in the SL and FF performance tests. In both groups (GH and GST), a 24-week intervention was necessary to see improvements in the SR tests as well as in the duration of the GH Bruce test.

These data show that a shorter intervention time is required to observe positive changes in the functional tests related to motor force and cardiorespiratory endurance. In tests where flexibility was predominant, only in the flexibility test of the posterior muscles of the thigh (SR) there was a significant improvement in the GH and GST groups at the end of the 24-week intervention. These results are in agreement with those of Bergamin et al. (2013), Tsourlou et al. (2006) and Barbosa et al. (2002), who observed improvements in the seat and reach test in elderly women after the implementation of a program of hydro aerobics and ST.

However, in the SR test where flexibility of the upper limbs prevailed, significant positive differences between T2 and T3 were only observed in the GH group, although no significant differences between experimental groups were observed. This may be due to the elderly subjects themselves who participated in this study, especially the subjects in the GST group, as many suffered from joint problems, a common problem in this age group. Moreover, scapulohumeral joint problems were not a basis for inclusion/rejection from this study.

Reinforcing the earlier idea, when we observed the reference values for the SR test presented by Jones and Rikli (2002), we found higher values at all times for the respective age groups in relation to the elderly subjects. In agreement with our results, Bergamin et al. (2013) suggested that hydro aerobic programs could positively impact flexibility of the lower limbs in elderly women. However, the use of stretching exercises in hydro aerobic sessions and not in the sessions of ST may have influenced the best performances between T2 and T3 in the measurement times of the GH group.

Relatively, for cardiorespiratory fitness measured over the Bruce test time duration, the GST group obtained significant improvements compared to the CG group. This result may be related to an increased motor skills performance and lower limb strength, observed indirectly through SS. The GST performance values were consistent with the study of Toraman et al. (2004), which demonstrated that a group of 42 elderly women aged between 60 and 86 years significantly improved aerobic endurance and lower limb strength after nine weeks of training that consisted of three weekly sessions of walking, strength training and flexibility. In addition, Ali et al. (2005) confirmed that an experimental elderly group, after an aerobic intervention of six months, significantly improved their cardiorespiratory capacity and quality of life. Schuch et al. (2013) presented similar results with regard to the improvement of the quality of life through water-based exercises.

Likewise, these data may indicate an improvement in efficiency and economy of cardiorespiratory fitness (Hartman et al., 2007). These results are in agreement with the data obtained by Brentano et al. (2008), who found positive and significant differences after the application of two types of ST for 24 weeks in the duration of the incremental test for the assessment of VO_2max_. They justify these results as peripheral adaptations arising from ST, in particular, the relative capillarization and an increase in enzymatic activity.

At the beginning of the program, the values for systolic and diastolic blood pressure in the GH and GST groups were normal values for that age range. No significant differences in the improvements in DBP and Systolic Blood Pressure (SBP) were observed in either group (GH and GST) at the end of 24 weeks of intervention. These results disagree with those of Colado et al. (2009b), who observed that the maintenance of SBP and DBP decreased in post-menopausal women who were subjected to 24 weeks of hydro aerobic or ST. In the aforementioned study, no changes in either DBP or SBP were observed in the control group. The results of our study and the study by Colado et al. (2009b) reinforce the idea presented by Cornelissen and Fagard (2005) in their meta-analysis on the effect of ST on blood pressure, where ST may function as a non-drug intervention for the reduction of DBP.

The importance of implementing exercise programs for postmenopausal women is evident from the results obtained in this work, especially when we consider the results of the control group. Nakamura et al. (2007) and Blankevoort et al. (2010) related functional autonomy to the level of fitness showing a direct relationship between these factors with social aspects or with the acquisition of healthy practices and habits that arise from day to day.

Bento et al. (2012) indicated that water activities, such as hydro aerobics, could be positive in relation to physical performance and functional capacity and that they could decrease the risk of falls in the elderly. The research of Simons and Andel (2006) and Seguin et al. (2008) pointed to an improvement in neuromuscular fitness and in cardiorespiratory fitness through the practice of strength training, while the study by Kimura et al. (2010) indicated that exercise programs without resistance were becoming an increasingly common method in the recommendation of activities to the elderly to improve their neuromuscular ability, functional autonomy and self-esteem. In this context, the water environment seems to be a great alternative for prescribing activities for the elderly, as the hydro aerobics were able to promote significant benefits in functional autonomy, neuromuscular capacity and cardiorespiratory variables in the elderly subjects in this study.

Thus, we consider the implementation of public policies aimed at the modification of the habits of younger individuals and the implementation of physical exercise for senior citizens, in terms of increasing their functional autonomy and quality of life, as important.

In conclusion, there is a positive effect of the practice of hydro aerobics and strength training on the cardiorespiratory and functional autonomy of post-menopausal women. Thus, a reinforcement of the efficacy of these two forms of exercise within this age group would be beneficial. There were significant differences between the strength training group and the hydro aerobic group with respect to the dependent variables: BC at T1, T2 and T3; BMI at T1, T2 and T3; SS at T3; SR and FF at T2 and moments in T2 and T3; leading us to believe that, overall, strength training was more effective than hydro aerobic training. The effect of activities in the GH showed significant difference in the dependent variables: SS at T2 and T3; SR at T3; GUW at T2 and T3; and DBP T_totaltest_ in T3, while the effect of activities of the GST was significantly different with respect to the dependent variables: SS at T2 and T3; SR at T3; GUW at T2 and T3; FF and DBP at T2 and T3; VO_2sub_ at T3; and T_totaltest_ at T2 and T3, supporting the idea that physical exercise programs promote improvements in some indicators of fitness, namely, functional and cardiorespiratory fitness of postmenopausal women in Portugal.

## Figures and Tables

**Table 1:** Description of the modified Bruce Protocol for a treadmill

**Stage**	**Duration (min)**	**Velocity (km/h)**	**Inclination (%)**
1	3	2.7	0.0
2	3	2.7	5.0
3	3	2.7	10.0
4	3	4.0	12.0
5	3	5.5	14.0
6	3	6.8	16.0

**Table 2a:** Mean values and standard deviations of the different study variables(CG and GH)

	**CG (n = 7)**			**GH (n = 17)**		

**Dependent Variables**	**T1 (Pre-Test)**	**T2 (12 weeks)**	**T3 (24 weeks)**	**T1 (Pre-Test)**	**T2 (12 weeks)**	**T3 (24 weeks)**
	
**BC (kg)**	73.20±10.85	72.20±10.00	73.48±10.88	75.11±6.88	74.29±6.16	74.92±5.67
**BMI (kg/m^2^)**	30.98±4.58	30.55±4.13	31.08±4.40	31.68±3.32	31.34±3.12	31.61±2.99
**SS (sets)**	13.50±6.56	13.50±7.55	14.75±5.12	17.81±4.23	21.56±4.32^*‡^	23.56±6.08^**‡^
**RBB (cm)**	−13.88±10.63	−19.00±5.87	−9.75±9.21	−17.88±14.15	−19.11±11.89	−10.41±11.97^†^
**SR (cm)**	−14.14±6.89	−12.30±5.87	−5.07±3.35	−10.21±5.79^‡^	−2.31±9.58	5.44±7.958^**†^
**GUW (s)**	7.32±3.03	6.91±2.22	6.22±1.81	5.48±1.16	5.05±1.01^*‡^	4.64±0.80^**†‡^
**FF (sets)**	16,50±3,70	16,75±5,74	16,75±6,40	21,50±3,20^‡^	21,63±5,25	22,37±6,38
**SBP (mmHg)**	143,57±16,99	140,38±14,37	141,44±24,31	134,94±13,52	124,80±32,24	120,08±27,81
**DBP (mmHg)**	71,43±9,43	78,57±13,56	69,28±5,53	72,41±8,89	71,00±8,89	66,42±10,28^**†^
**VO_2sub_ (ml.kg-1.min-1)**	17,35±3,08	18,44±5,60	21,37±2,91	24,52±3,67	25,21±3,02	27,83±5,29
**T_total_ (_test)_) (s)**	9,40±3,21	11,00±1,42	10,48±1,50	11,03±1,85	12,09±1,59	13,04±1,54^**†^

BC – Body Composition; BMI – Body Mass Index; SS – Sit and Stand on the Chair in 30 s; RBB – Test of Reaching Behind the Back With the Hands; SR – The Sit and Reach Test; GUW – Get Up and Walk Back to 2.44 m and Sit Test; FF – Forearm Flexion for 30 s Test; SBP – Systolic Blood Pressure; DBP – Diastolic Blood Pressure; VO_2sub_ – Oxygen Submaximal Consumption; T_totaltest_ – Total Duration of Modified Bruce Protocol; T1- first instance of measurements; T2 – second instance of measurements (after 12 weeks); T3- third instance of measurements (after 24 weeks);

* p<0.05 between T1 and T2;

** p<0.05 between T1 and T3;

† p<0.05 between T2 and T3;

‡ p<0.05 between GH and CG; μ p<0.05 between GST and CG; ¥ p<0.05 between GST and GH

**Table 2b:** Mean values and standard deviations of the different study variables(GST)

	**GST (n = 14)**		

**Dependent Variables**	**T1 (Pre-Test)**	**T1 (Pre-Test)**	**T1 (Pre-Test)**
	
**BC (kg)**	67.91±7.94^¥^	67.91±7.94^¥^	67.91±7.94^¥^
**BMI (kg/m^2^)**	28.58±2.95^¥^	28.58±2.95^¥^	28.58±2.95^¥^
**SS (sets)**	18.50±3.63	18.50±3.63	18.50±3.63
**RBB (cm)**	−7.54±9.79	−7.54±9.79	−7.54±9.79
**SR (cm)**	1.61±9.05 ^μ^	1.61±9.05 ^μ^	1.61±9.05 ^μ^
**GUW (s)**	5.00±0.67 ^μ^	5.00±0.67 ^μ^	5.00±0.67 ^μ^
**FF (sets)**	22,86±2,77 ^μ^	22,86±2,77 ^μ^	22,86±2,77 ^μ^
**SBP (mmHg)**	132,14±15,72	132,14±15,72	132,14±15,72
**DBP (mmHg)**	72,26±7,53	72,26±7,53	72,26±7,53
**VO_2sub_ (ml.kg-1.min-1)**	25,96±3,93	25,96±3,93	25,96±3,93
**T_total _(_test)_) (s)**	10,58±2,19	10,58±2,19	10,58±2,19

BC – Body Composition; BMI – Body Mass Index; SS – Sit and Stand on the Chair in 30 s; RBB – Test of Reaching Behind the Back With the Hands; SR – The Sit and Reach Test; GUW – Get Up and Walk Back to 2.44 m and Sit Test; FF – Forearm Flexion for 30 s Test; SBP – Systolic Blood Pressure; DBP – Diastolic Blood Pressure; VO_2sub_ – Oxygen Submaximal Consumption; T_totaltest_ – Total Duration of Modified Bruce Protocol; T1- first instance of measurements; T2 – second instance of measurements (after 12 weeks); T3- third instance of measurements (after 24 weeks);

* p<0.05 between T1 and T2;

** p<0.05 between T1 and T3;

† p<0.05 between T2 and T3;

‡ p<0.05 between GH and CG;

μ p<0.05 between GST and CG;

¥ p<0.05 between GST and GH
